# Wide-gamut plasmonic color filters using a complementary design method

**DOI:** 10.1038/srep40649

**Published:** 2017-01-13

**Authors:** Seon Uk Lee, Byeong-Kwon Ju

**Affiliations:** 1 Display and Nanosystem Laboratory, College of Engineering, Korea University, Seoul 136-713, Republic of Korea; 2 Display Laboratory, Samsung Display Co., LTD., Yongin 17113, Republic of Korea

## Abstract

Plasmonic color filters (PCFs) can acquire primary colors from non-polarized incident light through a two-dimensional arrangement of subwavelength holes. However, owing to the geometry of the 2D array, unintended secondary transmitted peaks derived from the higher-order modes of the surface plasmon resonance (SPR) lead to color cross-talk with the primary peaks. Herein, we propose a complementary design method for generating high-purity red, green, and blue (R/G/B) by combining the G/B filters of hole-arrays with the R filters of dot-arrays. Metallic dot-array filters, wherein the wavelength band under 575 nm was effectively blocked by the induction of peak broadening, operated as optical high-pass filters exhibiting pure red, and consequently widen the color gamut of PCFs by 30% without loss of luminance and color tunability. This harmonious combination promises to yield competitiveness for a next-generation color filter by enhancing the color reproducibility of plasmonic nanostructures.

The selective filtering of visible light by the periodic subwavelength hole arrays and the extraordinary optical transmission (EOT) by SPR have been fundamentals of PCFs^1^,^2^,^3^,^4^,^5^,^6^,^7^,^8^,^9^,^10^,^11^,^12^. Unlike the conventional pigment-based color filters that only represent a single color with a single material, the three primary colors in PCFs can be obtained simultaneously from identical materials by adjusting the period of the array^13^,^14^,^15^,^16^. Although PCFs have an outstanding color tunability, it has been pointed out that their optical efficiency is still not sufficient for replacing the pigment-based color filters^7^,^8^,^9^,^17^. In this study, we focused on determining a unique design for improving the color performance, and it started out by understanding the several issues of PCFs.

## Results

### Color cross-talk in 2D array plasmonic color filters

**Figure 1:**
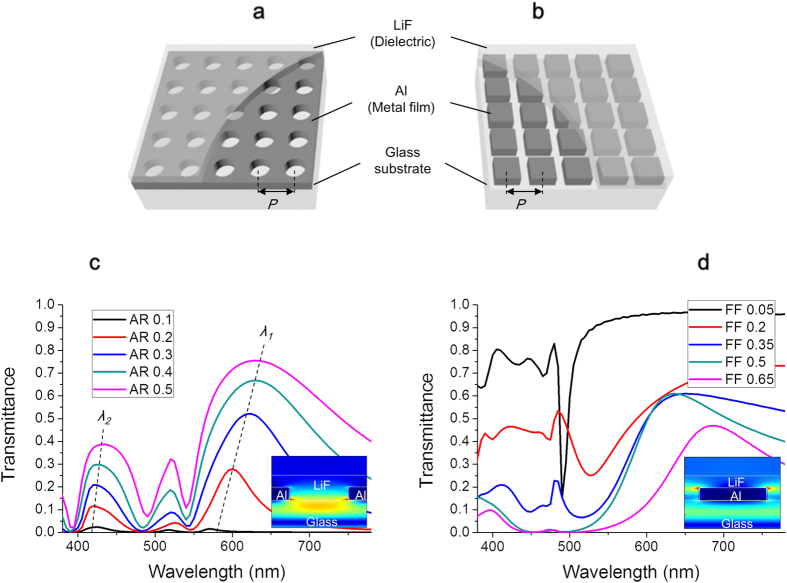
Peak-broadening phenomena in 2D square arrays of H-PCFs and D-PCFs. Schematic of the H-PCF (**a**) and the proposed D-PCF (**b**). The calculated transmission spectra of the H-PCFs (*P* = 360 nm), with ARs ranging from 0.1 to 0.5 (**c**) and of the D-PCFs (*P* = 330 nm), with FFs ranging from 0.05 to 0.65 (**d**). The insets of (**c**) and (**d**) illustrate the cross-sectional electric field profile at the maximum wavelength of 0.3 AR for the H-PCF and 0.5 FF for the D-PCF, respectively. FDTD software was used for modeling and simulations (see Methods).

Two types of spectral deformation can compromise the chromaticity. One is the existence of multiple transmitted peaks that cause color cross-talk. In the visible range, a single transmitted peak appears as a single color. If two or more peaks coexist, the additive mixing of different colors generates another color. Multiple peaks originate from the multiple modes of SPR and these modes can be determined by the geometry of the array. The relationship between the geometric parameters and the peak wavelength, λ_max_, in the square array is approximated by^2^,^10^


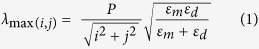


where *P* is the period of the array, *ε*_*m*_ and *ε*_*d*_ are the permittivities of the metal and the dielectric layer, respectively, and *i* and *j* are the scattering orders of the array. If the central wavelength of the 1^st^ order peak determined by the resonance mode (±1, 0) is *λ*_*1*_ and that of the 2^nd^ order peak determined by the resonance mode (±1, ±1) is *λ*_*2*_, the relationship 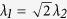 holds by (1). Subsequently, *λ*_*2*_ of the R/G/B filters with *λ*_*1*_ = 620 nm, 535 nm, and 440 nm becomes 438 nm, 378 nm, and 311 nm, respectively. In contrast to the green and blue, which belong to the relatively short-wavelength band, the 2^nd^ order peak of the red filter appears as a complete peak shape in the visible blue band (Fig. 1c). The blue light leakage in the red filter then shifts the color coordinates from red to magenta, which consequently reduces the color gamut of the R/G/B filters. In the same manner, it has been reported that the color cross-talk of red filters also occurs in hexagonal arrays^3^,^11^. So far, researchers have focused on lowering the color cross-talk, and were able to weaken the secondary peaks intensity or to increase the distance between the 1^st^ order peak and the 2^nd^ order peak^4^,^5^,^6^. However, the secondary peaks are yet to be removed.

### Peak-broadening and proposed dot-array designs for high saturated red

The other type of spectral deformation is peak-broadening that yields color degradation. Whereas the color cross-talk changes hue by the mixing of two or more different colors, the color degradation is related to the decrease in saturation. Although EOT contributes to enhancing the intensity, the hole-array PCFs (H-PCFs) with small openings, which are spatially segmented with a light-impermeable metal film, have inherently low transmittance^3^,^18^,^19^. Increasing the aperture ratio (AR = Area of the hole/Area of the lattice) for compensating for the low brightness results in a reduction in the selective filtering function that lowers the saturation^4^,^20^. This is observed in the form of peak-broadening, where both the height and width of the peak increase, as shown in Fig. 1c.

Now, we propose a new designing method to solve the aforementioned color cross-talk problem by using the peak-broadening characteristics of the PCFs. A paradoxical breakthrough was made possible by the introduction of dot-arrays, which are reversals of the hole-arrays. Since the periodic metallic dots play the role of a light-reflector, its reflection spectrum is shaped as peaks and its transmission spectrum is shaped as dips (inverted peaks)^9^,^16^,^21^,^22^,^23^. Such a spectrum, transmitting a wide range of wavelengths, is not favorable for the construction of additive R/G/B color filters. However, inducing peak-broadening of the inverted peaks by increasing the dot size will subsequently block most of undesirable light. As shown in Fig. 1d, at the period of 330 nm with low fill-factors (FFs = Area of the dot/Area of the lattice), the inverted peak appeared where the light of a specific wavelength was partially blocked. When the FF became higher than 0.5, the light in the 440–505 nm wavelength range was completely blocked and the mean transmittance in the 380–575 nm band decreased to less than 7%. This result implies that pure red extractable high-pass filters utilizing the dot-array PCFs (D-PCFs) can be designed by blocking short wavelengths. Consequently, if the dot-array-red (R_D_) PCF replaces the hole-array-red (R_H_) PCF causing a severe color cross-talk and is combined with the hole-array-blue (B_H_) and hole-array-green (G_H_) PCFs, highly saturated R/G/B PCFs are expected to be produced.

### A color gamut calculation of complementary plasmonic color filters

**Figure 2:**
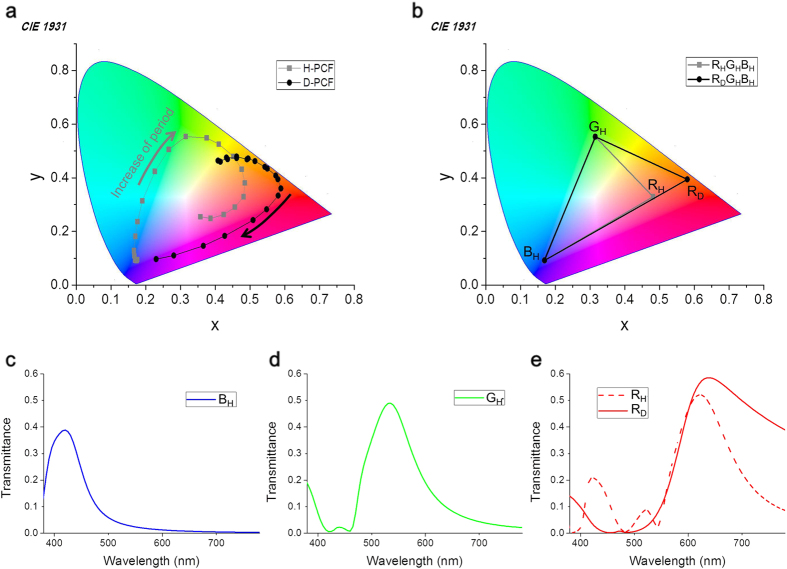
Colorimetric calculations for H-PCFs and D-PCFs. (**a)** The trend of calculated color coordinates (*x, y*), corresponding to the period in the 200–400 nm range. An AR value of 0.3 for the H-PCF and a FF value of 0.5 for the D-PCF were used for the two structures to be compared at a similar brightness. (**b)** The maximum color gamut illustrations of the R_H_G_H_B_H_ and R_D_G_H_B_H_ combinations. The periods of the individual filters were B_H_ = 220 nm, G_H_ = 300 nm, R_H_, = 360 nm, and R_D_ = 320 nm. The calculated transmission spectra of the B_H_ PCF (**c**), the G_H_ PCF (**d**) and the R_H_ PCF and R_D_ PCF (**e**).

In Fig. 2a, as the periods of the H-PCF and D-PCF increased from 200 nm to 400 nm in steps of 10 nm, the color coordinates of the former shifted along in the order of blue, green, and red, while those of the latter shifted from yellow, to red, and then to magenta. The colors that could be generated by the D-PCFs were limited compared with those that could be generated by the H-PCFs. In Fig. 2b, the maximum color gamut areas, calculated within each triangle, are 35 and 46% of the NTSC gamut for the R_H_G_H_B_H_ PCFs and R_D_G_H_B_H_ PCFs (complementary PCFs), respectively, while the mean luminance of R/G/B are 19% for the both PCFs. Thus, it can be expected that the combination of R_D_G_H_B_H_ enhances the color reproducibility by 31% without any loss of luminance. As expected, the spectral shape of the 2^nd^ order peak as in R_H_ is not observed in R_D_ (Fig. 2e). The mean transmittance in the 575–780 nm range of wavelengths is 48% for R_D_ and 30% for R_H,_ while that in the 380–575 nm range is 5.9% for R_D_ and 9.4% for R_H_, and the color coordinates (*x, y*) are (0.582, 0.395) for R_D_ and (0.483, 0.330) for R_H_. This means that R_D_ has a higher transmittance in the pass-band, a lower transmittance in the stop-band, and a higher saturation, compared with R_H_. We also conducted modelling and calculations for the other types of plasmonic structure, which are having a hexagonal lattice, or different materials. In terms of color expression, the complementary PCFs performed better than the H-PCFs in all cases (see Supplementary Information).

### Color generations of fabricated complementary plasmonic color filters

**Figure 3:**
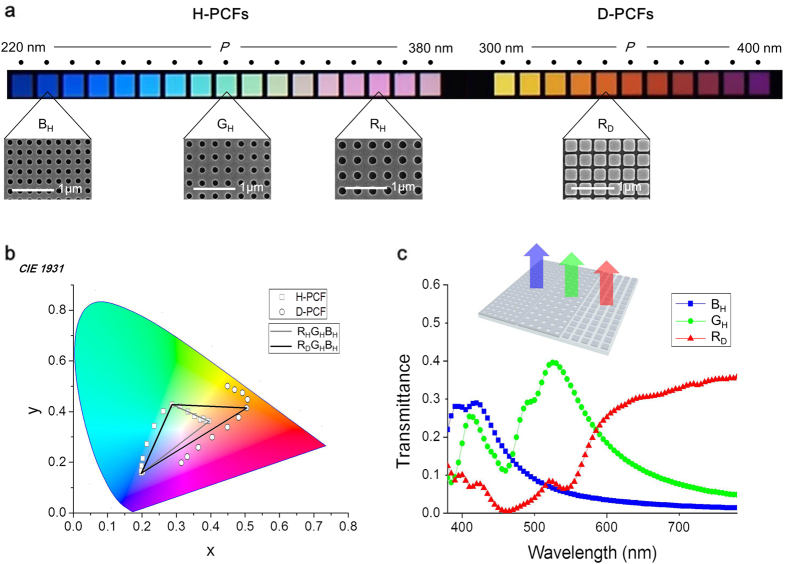
Demonstration of the H-PCFs and D-PCFs. (**a)** The optical microscopic and the scanning electron microscopic images of the fabricated PCFs. (**b**) The measured color coordinates (*x, y*) of the fabricated H-PCFs and D-PCFs, and the maximum color gamut illustrations for the R_H_G_H_B_H_ and R_D_G_H_B_H_ combinations. The periods of the individual filters are B_H_ = 230 nm, G_H_ = 300 nm, R_H_, = 360 nm, and R_D_ = 340 nm. (**c**) The measured transmission spectra of complementary PCFs.

We demonstrated the D-PCFs and H-PCFs by using the same materials and processes (see Methods). The H-PCF was designed to have 17 domains, with the period in the 220–380 nm range, in steps of 10 nm, while the design of the D-PCF included 11 domains with the period in the 300–400 nm range, in steps of 10 nm. The entire 28 domains were patterned simultaneously by using electron beam lithography. The area of a single domain was 40 μm × 40 μm. In common with the fabricated H-PCFs, the fabricated D-PCFs also indicated distinct colors from non-polarized incident light. As predicted by simulations, R_D_ PCF generated higher saturated red whereas R_H_ PCF yielded purplish-red (Fig. 3a). The transmittance at the peak wavelength and the color gamuts obtained from the measured color coordinates were reduced compared with the calculations, as shown in Fig. 3b, due to the surface reflection of glass substrates not considered in the simulation and the scattering caused by imperfections of fabrication: a wavy top surface by the step coverage of a dielectric layer near the cavity, grain boundaries and surface roughness of metal and dielectric layers formed by the thermal evaporation and dry etching, and nonuniformity of the thickness of layers and the shape and size of holes. But the results were sufficient for demonstrating the difference between the H-PCFs and complementary PCFs. The maximum color gamut of the R_D_G_H_B_H_ combination was 17% of the NTSC gamut, whereas that of the R_H_G_H_B_H_ combination was only 10% of the NTSC gamut. In Fig. 3c, the peak transmittance of R_D_, G_H_, and B_H_ was in the 29–40% range and the mean luminance was 16%.

## Discussion

While there have been numerous studies on nanostructures employing either hole-arrays or dot-arrays, the synergistic effect achieved from combining the two was more remarkable. The H-PCFs were able to express a wide range of colors, but exhibited a weak red color, owing to the color cross-talk. In contrast, the D-PCFs were not able to generate all of the three primary colors, but exhibited a strong red color. Most importantly, the harmony of complementary PCFs was able to maintain the strong advantage of color tunability, since both the two different PCFs have been implemented by using an identical layer-structure.

## Methods

### Simulation

The 3D finite-difference time-domain (FDTD, Lumerical Solutions) software was used for the simulation. Both the H-PCFs and the D-PCFs were modeled, wherein the glass substrates were coated with a 130-nm-thick metal layer of Al, followed by a 110-nm-thick dielectric layer of LiF. The Al film of the H-PCF was perforated with subwavelength circular holes, and that of the D-PCF was patterned with subwavelength square dots. The mesh scale for each of d*x*, d*y*, and d*z* was 5 nm with a staircase approximation. A plane-wave type light source was used for the simulation.

### Fabrication

For the substrate, a 0.5-mm Eagle XG glass (Corning) was used. A 130-nm-thick Al layer was thermally evaporated on the glass substrate. An electron beam resist (ZEP-520a, Zeon Chemicals) was coated by spin coating for patterning. The hole-arrays and dot-arrays were patterned simultaneously in an e-beam lithography (JBX-9300FS, JEOL) process. Next, Cl_2_-based inductive coupling plasma reactive ion etching (ICP-RIE, Plasmalab System 100, Oxford Instruments) was performed to etch the Al film. The resist residue was removed by using a remover (ZDMAC, Zeon Chemicals) after the Al etching. Finally, a 110-nm-thick LiF layer was deposited by thermal evaporation.

### Optical measurement

The transmitted spectra were measured by using a LCF-2100 (Otsuka Electronics) spectrophotometer, with the measuring spot size of 10 μm. The optical image was obtained by using an optical microscope, DM-6000-M (Leica Microsystems).

## Additional Information

**How to cite this article**: Lee, S. U. and Ju, B.-K. Wide-gamut plasmonic color filters using a complementary design method. *Sci. Rep.* **7**, 40649; doi: 10.1038/srep40649 (2017).

**Publisher's note:** Springer Nature remains neutral with regard to jurisdictional claims in published maps and institutional affiliations.

## Supplementary Material

Supplementary Information
