# Supplementary material for: Wide-gamut plasmonic color filters using a complementary design method
Source: Sci Rep. 2017 Jan 13;7:40649. doi: 10.1038/srep40649 (PMC5234004; doi:10.1038/srep40649)
Supplement: Supplementary Information [file srep40649-s1.pdf]

Wide-gamut plasmonic color filters using a complementary design method

Seon Uk Lee[1,2] and Byeong-Kwon Ju[1,*]

[1]Display and Nanosystem Laboratory, College of Engineering, Korea University, Seoul 136-713, Republic of Korea
[2]Display Laboratory, Samsung Display Co., LTD., Yongin 17113, Republic of Korea
* Corresponding author : bkju@korea.ac.kr

□ Calculations for the other types of plasmonic structure.

We also conducted simulations for the other types of plasmonic structure, which are having a hexagonal lattice, or different materials. In terms of color expression, the complementary PCFs (combination of $R_DG_HB_H$) performed better than the hole-array PCFs (combination of $R_HG_HB_H$) in all cases. The complementary PCFs are expected to enhance the color reproducibility by 21~45% without loss of luminance.

Case 1) The plasmonic structure having an index matching layer.

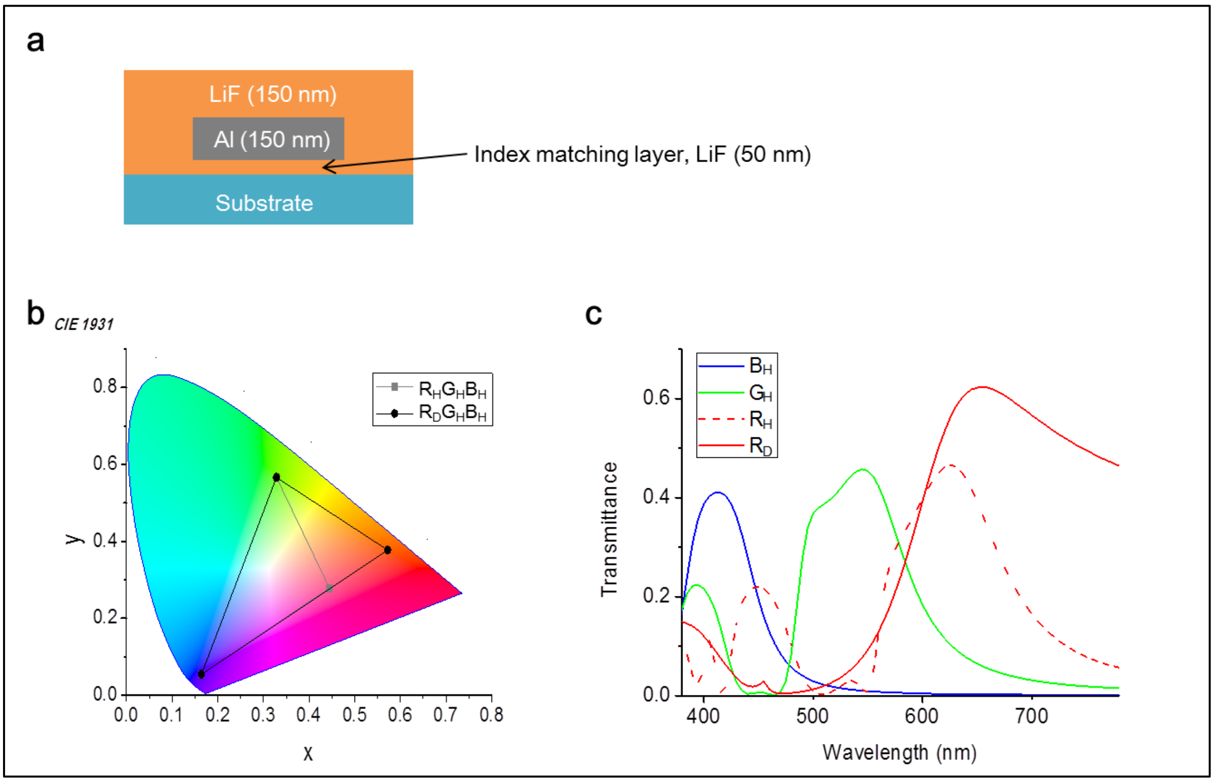

Figure S1. Calculations for the plasmonic structure having an index matching layer.

a, Schematic of the structures. **b,** The maximum color gamut illustrations for the $R_HG_HB_H$ and $R_DG_HB_H$ combinations. **c,** The calculated transmission spectra of B_H, G_H, R_H, and R_D

Table S1. Calculated color coordinates and color gamuts.

<table>
<tr><th></th><th>Period
(nm)</th><th>Hole diameter
Dot size*
(nm)</th><th>x</th><th>y</th><th>Y</th><th>$R_HG_HB_H$
Mean luminance
(%)
Color gamut
(% of NTSC)</th><th>$R_DG_HB_H$
Mean luminance
(%)
Color gamut
(% of NTSC)</th></tr>
<tr><td>R_H</td><td>370</td><td>229</td><td>0.445</td><td>0.279</td><td>17.3</td><td rowspan="3">17.1
33.9</td><td></td></tr>
<tr><td>G_H</td><td>310</td><td>192</td><td>0.328</td><td>0.567</td><td>32.6</td><td rowspan="3">17.3
49.3</td></tr>
<tr><td>B_H</td><td>220</td><td>136</td><td>0.164</td><td>0.055</td><td>1.5</td></tr>
<tr><td>R_D</td><td>310</td><td>210*</td><td>0.572</td><td>0.378</td><td>17.9</td><td></td></tr>
</table>

Case 2) The plasmonic structure having a SiO_2 dielectric layer.

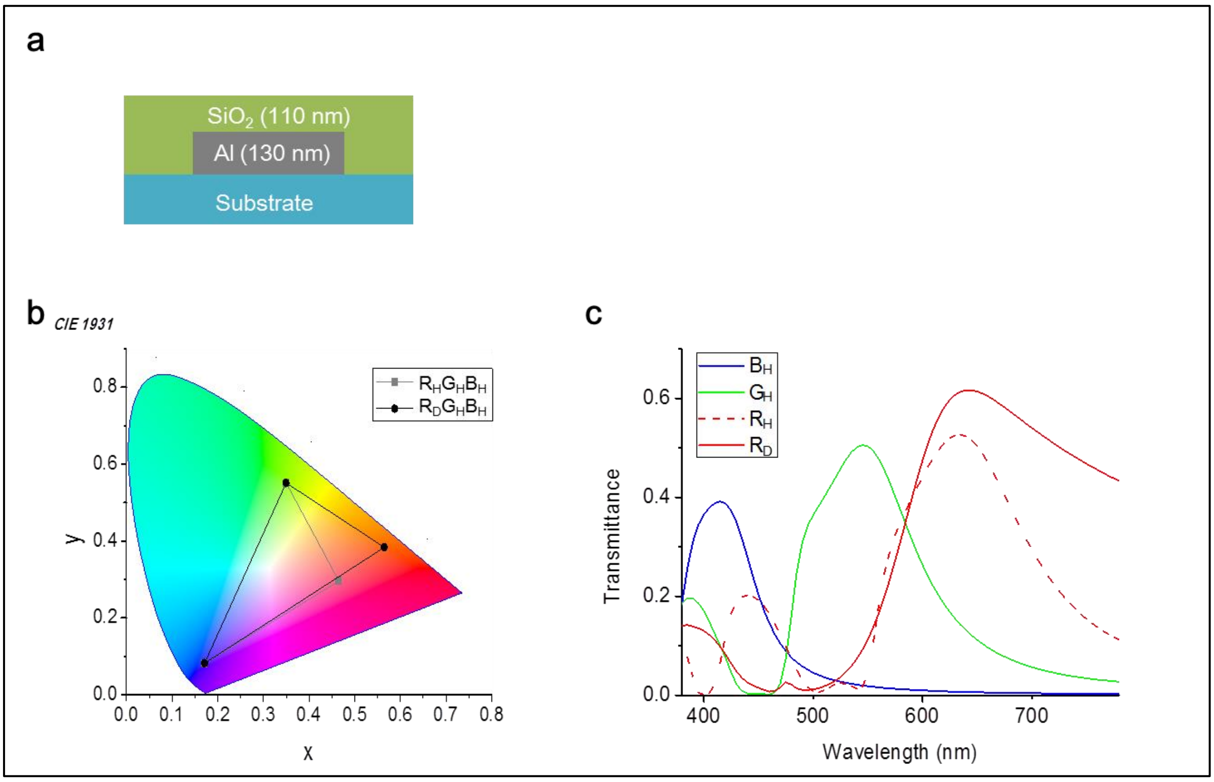

Figure S2. Calculations for the plasmonic structure having a SiO_2 dielectric layer.

a, Schematic of the structures. **b,** The maximum color gamut illustrations for the $R_HG_HB_H$ and $R_DG_HB_H$ combinations. **c,** The calculated transmission spectra of B_H, G_H, R_H, and R_D

Table S2. Calculated color coordinates and color gamuts.

<table>
<tr><th></th><th>Period (nm)</th><th>Hole diameter
Dot size*
(nm)</th><th>x</th><th>y</th><th>Y</th><th>R$R_HG_HB_H$
Mean luminance (%)
Color gamut (% of NTSC)</th><th>$R_DG_HB_H$
Mean luminance (%)
Color gamut (% of NTSC)</th></tr>
<tr><td>R_H</td><td>360</td><td>222</td><td>0.465</td><td>0.297</td><td>19.7</td><td rowspan="3">20.0
31.5</td><td></td></tr>
<tr><td>G_H</td><td>300</td><td>185</td><td>0.350</td><td>0.552</td><td>37.9</td><td rowspan="3">20.5
41.4</td></tr>
<tr><td>B_H</td><td>210</td><td>130</td><td>0.171</td><td>0.083</td><td>2.4</td></tr>
<tr><td>R_D</td><td>320</td><td>224*</td><td>0.565</td><td>0.384</td><td>21.1</td><td></td></tr>
</table>

Case 3) The plasmonic structure having a hexagonal lattice.

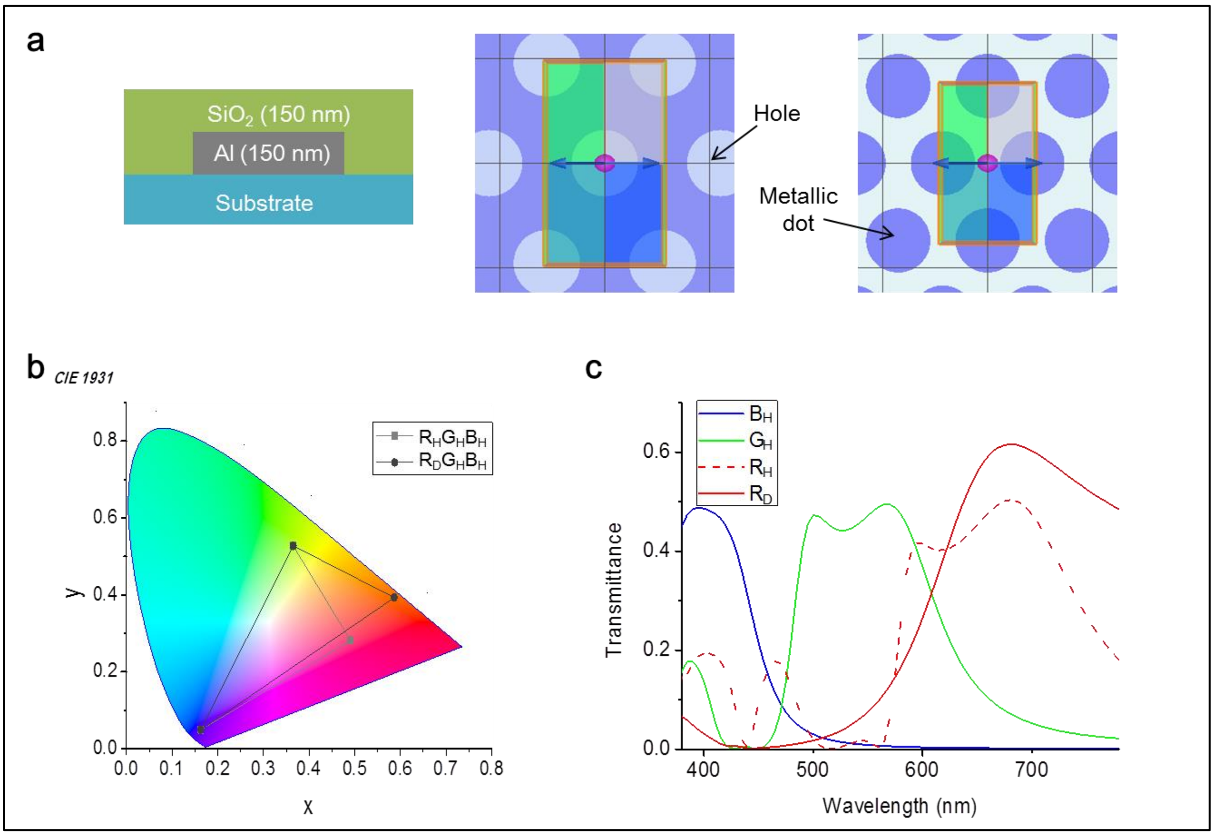

Figure S3. Calculations for the plasmonic structure having a hexagonal lattice.

a, Schematic of the structures. **b,** The maximum color gamut illustrations for the $R_HG_HB_H$ and $R_DG_HB_H$ combinations. **c,** The calculated transmission spectra of B_H, G_H, R_H, and R_D

Table S3. Calculated color coordinates and color gamuts.

<table>
<tr><th></th><th>Period
(nm)</th><th>Hole diameter
Dot diameter*
(nm)</th><th>x</th><th>y</th><th>Y</th><th>$R_HG_HB_H$
Mean luminance
(%)
Color gamut
(% of NTSC)</th><th>$R_DG_HB_H$
Mean luminance
(%)
Color gamut
(% of NTSC)</th></tr>
<tr><td>R_H</td><td>440</td><td>253</td><td>0.490</td><td>0.283</td><td>13.8</td><td rowspan="3">18.9
34.6</td><td></td></tr>
<tr><td>G_H</td><td>350</td><td>201</td><td>0.365</td><td>0.528</td><td>41.4</td><td rowspan="3">18.8
42.0</td></tr>
<tr><td>B_H</td><td>230</td><td>132</td><td>0.162</td><td>0.050</td><td>1.4</td></tr>
<tr><td>R_D</td><td>330</td><td>245*</td><td>0.586</td><td>0.394</td><td>13.5</td><td></td></tr>
</table>
